# Imprinting superconducting vortex footsteps in a magnetic layer

**DOI:** 10.1038/srep27159

**Published:** 2016-06-06

**Authors:** Jérémy Brisbois, Maycon Motta, Jonathan I. Avila, Gorky Shaw, Thibaut Devillers, Nora M. Dempsey, Savita K. P. Veerapandian, Pierre Colson, Benoît Vanderheyden, Philippe Vanderbemden, Wilson A. Ortiz, Ngoc Duy Nguyen, Roman B. G. Kramer, Alejandro V. Silhanek

**Affiliations:** 1 Université de Liège, Département de Physique, Sart Tilman, B-4000, Belgium; 2 Universidade Federal de São Carlos, Departamento de Física, São Carlos, 13565-905 SP, Brazil; 3 Université Grenoble Alpes, Institut NEEL, F-38000 Grenoble, France; 4 CNRS, Institut NEEL, F-38000 Grenoble, France; 5 Université de Liège, Center for Applied Technology in Microscopy (GREENMAT), Sart Tilman, B-4000, Belgium; 6 Université de Liège, SUPRATECS and Department of Electrical Engineering and Computer Science, Sart Tilman, B-4000, Belgium

## Abstract

Local polarization of a magnetic layer, a well-known method for storing information, has found its place in numerous applications such as the popular magnetic drawing board toy or the widespread credit cards and computer hard drives. Here we experimentally show that a similar principle can be applied for imprinting the trajectory of quantum units of flux (vortices), travelling in a superconducting film (Nb), into a soft magnetic layer of permalloy (Py). In full analogy with the magnetic drawing board, vortices act as tiny magnetic scribers leaving a wake of polarized magnetic media in the Py board. The mutual interaction between superconducting vortices and ferromagnetic domains has been investigated by the magneto-optical imaging technique. For thick Py layers, the stripe magnetic domain pattern guides both the smooth magnetic flux penetration as well as the abrupt vortex avalanches in the Nb film. It is however in thin Py layers without stripe domains where superconducting vortices leave the clearest imprints of locally polarized magnetic moment along their paths. In all cases, we observe that the flux is delayed at the border of the magnetic layer. Our findings open the quest for optimizing magnetic recording of superconducting vortex trajectories.

Quantum magnetic flux units in type-II superconductors can be regarded as small solenoids, producing localized magnetic field variations extending over distances of 2*λ* perpendicularly to the applied field, where *λ* is the magnetic penetration depth. These flux lines interact with inhomogeneities *inside* the superconducting material, leading to a plethora of well-known pinning mechanisms. Furthermore, the static and dynamic response of individual vortices can also be modified if their stray field *outside* the superconducting volume is altered. This is precisely the reason why multilayered hybrid systems combining superconducting and non-superconducting materials have been an active field of research in the last decade.

It has been shown, for instance, that the stray field of an isolated moving vortex can induce eddy currents in a nearby metallic layer, leading to a velocity dependent damping force^1^. This effect can be exploited to provide better stability and quench protection in superconducting cables by reducing the speed of flux jumps^3^. More spectacular is the possible use of the monopolar magnetic field emanating from a vortex as a submicron-sized magnetic tweezers to locally polarize spin carriers in a diluted magnetic semiconductor^5^. Among these hybrid devices, the heterostructures combining superconductivity and magnetism, two antagonistic quantum macroscopic states, arguably represent the most investigated systems so far^6^,^7^,^8^,^9^.

A simple attempt to classify the superconductor (S-layer) - ferromagnet (F-layer) hybrids, where the F-layer and the S-layer are in close proximity and interact only through their stray fields, consists of comparing the relative field strength scales of each subsystem. Indeed, if the coercive field, *H*_coe_, of the ferromagnetic layer is much larger than the upper critical field, *H*_c2_, of the superconducting layer, changing the magnetic landscape in the S-layer will have no major effect on the F-layer. In contrast to that, modifications in the F-layer can lead to profound changes in the response of the S-layer. This effect has been successfully implemented to obtain fluxonic elemental devices such as tunable pinning centres and vortex rectifiers^10^,^11^,^12^. In the opposite case where *H*_coe_ ≲ *H*_c2_, i.e., combining a hard type-II superconductor and a soft magnetic material, it has been shown that magnetic domains in the F-layer shrink compared to their size without the superconducting layer^13^,^14^,^15^ and that vortex-antivortex lattices can be spontaneously induced^16^,^1^7 when cooling down in zero applied field below the superconducting critical temperature *T*_c_.

A particularly interesting phenomenon, previously evoked to explain the increase of pinning in Nb with ferromagnetic Gd inclusions, is the possibility to locally polarize a ferromagnetic material by the stray field of a single vortex^18^. If this were indeed feasible, we could imagine a single vortex as a tiny magnetic scriber leaving a clear imprint, consisting of locally polarized magnetic moments, on the magnetic material. It is precisely this possibility of using vortices as magnetic tweezers that we experimentally investigate in the present manuscript via the magneto-optical imaging technique. Our hybrid system consists of a superconducting Nb film separated from a permalloy (Py) film by a thin insulating SiO_2_ layer (see Fig. 1). The Py layer only covers partially the Nb film, thus allowing us to compare the behaviour of flux penetration in the Nb layer without and with a soft magnet on top. We demonstrate that regardless of the Py thickness, a net in-plane polarization of the F-layer introduces an asymmetry in flux penetration in the Nb layer. This magnetic landscape also influences the much faster flux motion in thermomagnetic instabilities. Thick Py layers show reorientable stripe domains that can guide the flux motion as reported by Vlasko-Vlasov *et al*.^19^. For thin Py layers without stripe domains, we observe that the flux in the superconductor leaves clear imprints on the Py layer. This effect is particularly prominent for vortex avalanches. Our findings extend recent reports on YBCO/CoFeB bilayers by Stahl *et al*.^20^, where mapping of the supercurrent flow on the magnetic layer is shown. Given the fact that the Curie temperature of Py is much higher than the critical temperature of the superconductor, the footsteps left by vortices on the Py layer can survive up to room temperature, thus allowing for *ex situ* analysis as in magnetic decoration techniques. This is further confirmed by imprinting a hard magnetic template into the Py layers at room temperature.

## Results and Discussion

**Figure 1:**
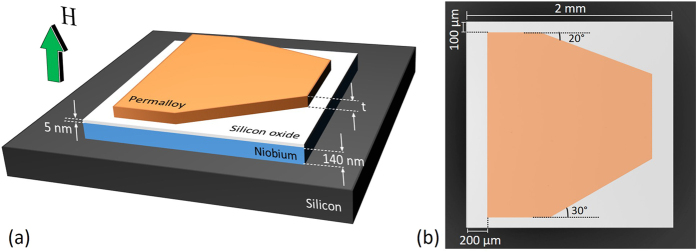
Hybrid system layout. (**a**) Schematic representation of the sample layout. For the magneto-optical images, a magnetic field *H* is applied perpendicularly to the plane of the sample. Three thicknesses *t* of the Py layer were investigated: 50 nm, 100 nm, and 460 nm. Panel (**b**) shows an artificially coloured top-view optical image of one of the samples.

The investigated hybrid system is composed of a Py (Fe_19_Ni_81_) polygonal-shaped layer (F) of three different thicknesses *t* = 50 nm, 100 nm and 460 nm, deposited on top of a 140 nm-thick Nb film (S) of 2 × 2 mm^2^ (see Fig. 1). A 5 nm-thick SiO_2_ film was deposited between the S and F layers to ensure electrical insulation between them, and thus reduced proximity effects. In other words, the mutual interaction between the S and F layers is purely of magnetic origin and occurs through their respective stray fields. The polygonal shape of the Py layer aims to explore different angles of penetration of the magnetic flux entering through the sides of the Nb square. In addition, the fact that the Py does not cover the Nb borders allows flux avalanches to develop freely. Details concerning sample preparation are provided in the Methods section. Magnetisation measurements reveal a superconducting transition *T*_c_ = 9 K and show that flux avalanches of thermomagnetic origin develop below 4.5 K, whereas flux penetration in the Nb layer is rather smooth above this temperature, where it can be properly described by the isothermal Bean model. Direct visualization of the magnetic flux landscape was obtained by magneto-optical imaging (MOI) (see Methods).

### Magnetic properties of the Py layer

Let us first consider the magnetic behaviour of soft magnetic alloys of the type Fe-Ni, known as permalloy. These compounds are characterized by a weak uniaxial anisotropy energy *K*, implying broad domain wall width 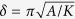, where *A* is the exchange constant, proportional to the coupling between neighbouring spins. Broad domain walls are relatively easy to displace and, therefore, magnetisation loops of these materials exhibit rather small irreversibility. Ni-rich permalloy compounds, such as the one used in this work, have a perpendicular anisotropy that dominates over shape anisotropy imposed by the thin film geometry above a critical thickness *t*_c_ ~ 200 nm^21^,^22^,^23^,^24^,^25^,^26^. Increasing the film thickness above *t*_c_ leads to out-of-plane up and down stripe domains separated by a Bloch-type domain wall^27^,^28^,^29^. It has been shown^30^ that the domain structure for thicknesses smaller than *t*_c_ corresponds to large domains with antiparallel in-plane magnetisation separated by broad Néel or cross-tie domain walls. More recently, however, Uspenskaya *et al*.^31^ have reported that Py deposited on top of a Nb film thicker than 100 nm, as in our experiments, can exhibit Bloch head-to-head domain walls which the authors attribute to the roughness-induced anisotropy.

**Figure 2:**
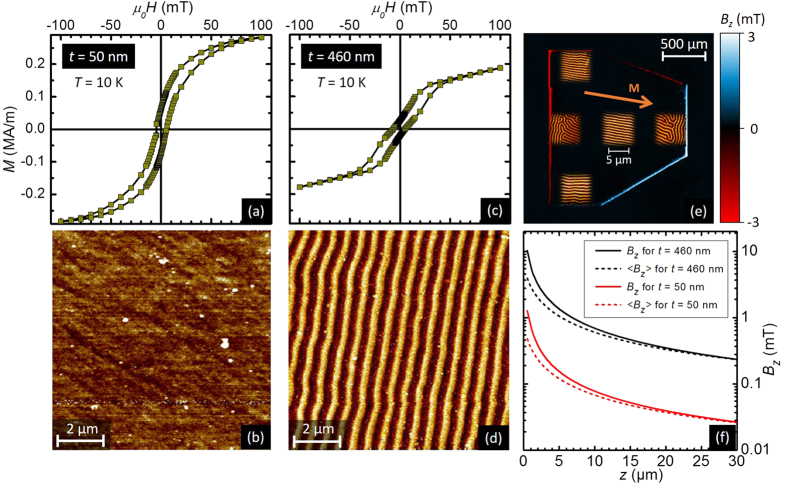
Magnetic characterization of the Py layers. Out-of-plane magnetisation *M* as a function of the external magnetic field *H*, applied perpendicularly to the plane of the sample (see Fig. 1(a)), obtained at 10 K for a Py layer of (**a**) 50 nm, and (**c**) 460 nm. Panels (**b,d**) show MFM images obtained at room temperature for the 50 nm and 460 nm-thick Py layers, respectively. (**e**) MO image of the stray field arising from the macroscopic in-plane magnetic moment (orange arrow) in the polarized 460 nm-thick Py layer. The insets show MFM images of the magnetic domains at various positions on the Py. (**f**) Numerical simulations show the decay of the magnetic field *B*_*z*_ at the border of the Py layer as a function of the vertical distance *z* from the top surface of Py (solid lines). The calculations give values of 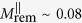 MA/m, based on the experimental mean magnetic field picked up by MOI, i.e. the average of the field 〈*B*_*z*_〉 over the garnet thickness *d* = 3 *μ*m (dashed lines).

Figure 2 summarizes the most interesting results of the magnetic characterization of these hybrid samples. Since the 50 nm-thick and the 100 nm-thick Py samples exhibit very similar behaviour, we will only focus on the two extreme thicknesses in the remainder of this work. Panels (a) and (c) show out-of-plane magnetisation loops at 10 K for an external field *H* applied perpendicularly to the sample. The remanent magnetization *M*_rem_ ≈ 0.08 MA/m in the 50 nm-thick Py is significantly larger than in the 460 nm-thick Py layer, where *M*_rem_ ≈ 0.025 MA/m. However, *M*_rem_ can be erased in both samples by applying a rather small field 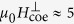 mT. The *M*(*H*) loops show little change at 300 K, with analogous values for the characteristic fields.

In agreement with previous reports^27^, magnetic force microscopy (MFM) images at room temperature show no magnetic contrast for Py films thinner than 100 nm (Fig. 2(b)). The thicker Py layer exhibits a stripe domain structure (Fig. 2(d)) which can be readily oriented with an in-plane magnetic field of a few mT. The stripes have a width *w* ≈ 280 nm. The contrasting magnetic landscapes observed in the thin and the thick Py layers give rise to remarkable and non-trivial differences in flux penetration, that will be addressed in the following sections.

The observed magnetic history of the Py layers leads us to adopt a clear measurement protocol. Starting from a temperature *T* = 10 K just above the superconducting transition, we then apply an in-plane magnetic field in such a way to clearly define the direction of the stripe domains as those illustrated in Fig. 2(d). This procedure not only orients the stripe domains, but also induces a macroscopic in-plane magnetic moment. Indeed, Fig. 2(e) shows a magneto-optical (MO) image of the 460 nm-thick Py layer at 10 K after magnetizing it along the direction indicated by the orange arrow. The blue-white and red stripes outlining the borders of the Py correspond to *B*_*z*_, the component of the stray field perpendicular to the sample plane. White indicates a stray field in the same direction as the applied field (i.e. positive field) and red indicates a stray field antiparallel to the applied field (i.e. negative field). Complementary MFM images obtained at remanence and over different places of the Py layer are also shown in Fig. 2(e). Interestingly, the stripe domains align mostly parallel to the previously applied in-plane field everywhere in the Py layer, except at distances of about 5 *μ*m from the borders perpendicular to the magnetisation. Measurements of the in-plane magnetization show a saturation magnetisation 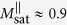 MA/m. In contrast to the case of perpendicular saturation magnetization, the in-plane saturation magnetization 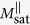 can be reached by applying small in-plane fields of around 15 mT for the thick Py, and 2 mT for the thin Py layer. However, fields around 3 mT (1 mT) were enough to change the direction of the in-plane magnetization in the thick (thin) Py.

Quantitative MOI analysis allows us to estimate the mean out-of-plane component of the magnetic stray field *B*_*z*_ at the extremes of the Py layer. Since *B*_*z*_ decays as the distance *z* measured from the top of the Py surface increases, each slice of the MO indicator will give a different contribution to the MO signal. Therefore, the experimentally picked up signal, shown in Fig. 2(e), corresponds to the average *z*-component of the stray field in the MO indicator:


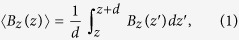


where *d* = 3 *μ*m is the thickness of the MO indicator. From Fig. 2(e), we found 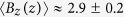 mT (0.35 ± 0.2 mT) at 10 K in the thick (thin) Py layer for a typical gap between the sample and the MO indicator of *z* ~ 1 *μ*m. It is then possible to calculate the remanent magnetisation 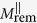 of the magnetic layer, based on the measured 〈*B*_*z*_(*z*)〉. To that end, we carried out numerical calculations for Py layers respecting exactly the same dimensions as the investigated samples, based on the following equation:


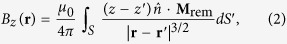


where *S* is the total surface of the magnetic layer and 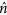 is the unit vector perpendicular to the surface. Combined with Eq. (1), Eq. (2) links 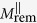 with 〈*B*_*z*_(*z*)〉. Using the experimental values for 〈*B*_*z*_(*z*)〉 and *z*, we find 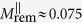 MA/m (0.08 MA/m) for the thick (thin) Py layer. The decay of *B*_*z*_(*z*) (solid lines) and 〈*B*_*z*_(*z*)〉 (dashed lines) with increasing *z* is shown in Fig. 2(f) for the 460 nm-thick Py (black) and for the 50 nm-thick Py (red) and were found to be very similar to the analytical solution valid for a parallelepiped magnetic layer^32^. Note that we considered a constant in-plane magnetisation all over the Py layer, while in reality, as appears clearly in Fig. 2(e), the magnetization may change direction close to the borders to reduce the stray field, thus leading us to underestimate 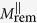.

**Figure 3:**
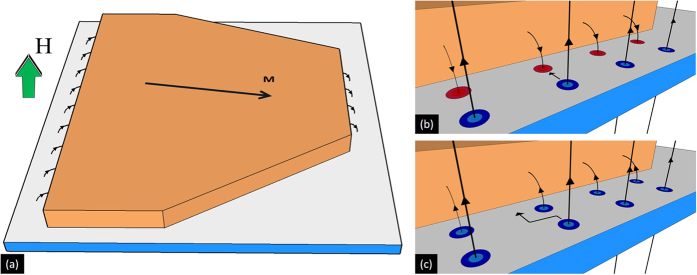
Interaction between vortices created by the Py layer and the applied field. (**a**) Sketch representing the stray field induced by the Py layer in the superconductor. When cooling down below *T*_c_, stray field flux lines are trapped in the Nb film and (**b**) antivortices (coloured in red) and (**c**) vortices (in blue) are generated at the Py borders perpendicular to the in-plane magnetisation. In (**b**), the antivortices attract and annihilate entering vortices created by the applied field, while in (**c**), both types of vortices interact repulsively.

The simulations suggest that the stray magnetic field at the Nb layer will reach much higher values than those detected by the garnet. Indeed, Fig. 2(f) shows that *B*_*z*_ is above 10 mT (1 mT for the thin Py) at the surface of the Py layer, which corresponds also to the field at the top surface of the superconductor. Note that the stray field polarity in the superconductor is reversed compared to the one detected by the magneto-optical indicator, as it is indicated in the sketch of Fig. 3(a). The question now arises as to whether the magnetic charge and the associated stray field is large enough to induce vortices of opposite polarities at the extremes of the Py layer. Milošević and Peeters^33^ have addressed this problem within the London formalism and they have shown that the number of vortices induced by the stray field depends on the ratio between the in-plane magnetisation *M* and *M*_0_ = 4*π*Φ_0_*λ*/*μ*_0_ ~ 1 MA/m for Nb at zero temperature. The high values of 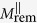 we calculated guarantee the creation of vortices at the extremes of the Py layer. Moreover, values of *B*_*z*_ at the superconductor surface are much higher than the typical values for the first vortex penetration.

### Anisotropic flux penetration: thick Py layer

**Figure 4:**
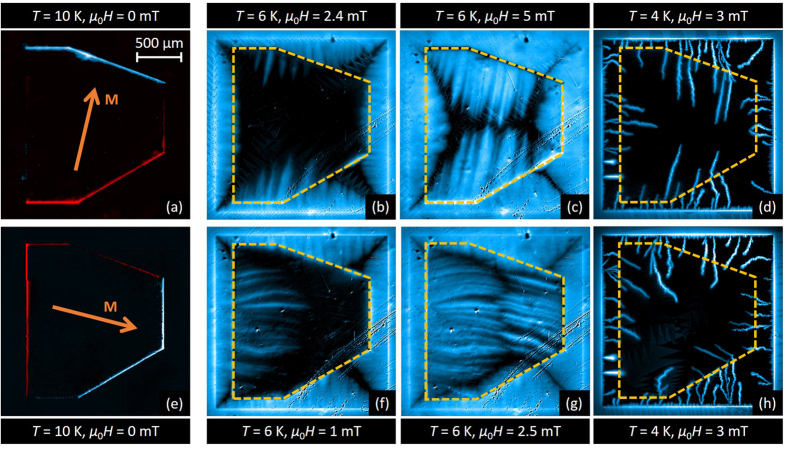
Highly anisotropic flux penetration into a Nb film covered with a 460 nm-thick Py layer. MO images of a Nb film with a thick (460 nm) Py layer after applying an in-plane field of 10 mT along a direction close to the vertical (upper row) and horizontal (lower row) axes. First column: images obtained at *T* = 10 K > *T*_c_. The white (red) rims evidence the positive (negative) stray field at the borders of the Py layer. Second and third column: MO images obtained at *T* = 6 K in the smooth flux penetration regime, for an applied field pointing out of the images, (**b**) *μ*_0_*H* = 2.4 mT, (**c**) *μ*_0_*H* = 5 mT, (**f**) *μ*_0_*H* = 1 mT, (**g**) *μ*_0_*H* = 2.5 mT. Last column: MO images taken at *T* = 4 K and *μ*_0_*H* = 3 mT in the thermomagnetic instabilities regime.

Having identified the magnetic landscape imposed by the Py layer, we are now in a position to explore its influence on flux penetration in the underlying superconducting layer. Figure 4 summarizes the most salient results for the hybrid system with a 460 nm-thick Py layer, for two nearly perpendicular directions of in-plane polarization as shown in panels (a) and (e). The first column images (panels (a) and (e) in Fig. 4) were obtained at *T* = 10 K > *T*_c_ in the remanent state. Subsequently, the sample was cooled down to 6 K and the out-of-plane magnetic field was increased in steps of 0.1 mT. Figure 4(b) corresponds to *μ*_0_*H* = 2.4 mT and Fig. 4(f) corresponds to *μ*_0_*H* = 1 mT. Under these zero-field-cooling conditions, the flux penetrates from the borders of the superconductor and the flux front progresses smoothly until it reaches the border of the Py layer, indicated by a yellow dashed line. Increasing *H* further leads to a distinctive field penetration into the region covered by the Py layer. Indeed, firstly, the flux front progression is arrested at the side of the Py layer generating a negative field in the Nb layer (top side in (b), right side in (f)), whereas it easily crosses the Py border that creates a positive field in the Nb layer (bottom side in (b), left side in (f)).

This behaviour can be explained by the fact that antivortices (vortices) are created at the white (red) border of the Py layer as illustrated in Fig. 3(b,c). Indeed, incoming vortices first annihilate with antivortices before moving further into the area covered by the magnetic layer (top side in Fig. 4(b), right side in (f)), as shown in Fig. 3(b). The antivortices at the Py border therefore act as attractive pinning centres for the vortices. On the contrary, vortices entering from the side where the Py layer induces vortices of the same polarity (Fig. 3(c)), find just a permeable wall of repulsive centers created by the vortices generated by the Py layer, thus not impeding flux penetration beneath it (bottom side in Fig. 4(b), left side in (f)). As expected, when a negative field *H* is applied, the sides of the Py layer where flux penetration is eased or delayed are inverted. Once the flux is pushed inside, it is guided by the stripe domains, giving rise to clear finger like magnetic structures, resembling those reported by Vlasko-Vlasov *et al*.^19^. It is interesting to note that a somewhat similar asymmetry in flux penetration has been discussed by Vodolazov *et al*.^34^ in micrometer scale structures. In addition, at the sides perpendicular to the in-plane magnetic moment of the Py layer (i.e. left and right borders in (b), upper and lower borders in (f)), field penetration is also delayed. This can be explained by the strong reduction of the vortex mobility perpendicular to the stripe domains^35^,^3^6. Although similar features in the highly anisotropic field penetration are observed in panels (b) and (f), the effect is less prominent in (b) due to the particular geometrical shape of the Py layer. Indeed, firstly the distance between the Py layer and the Nb border where the penetration occurs is twice smaller in (b) compared to (f). Secondly, the angle between the Py and the Nb borders is larger for the bottom side of the sample than for the top side, thus increasing the distance that flux has to cover before reaching the Py border.

Further increasing the field (panels (c) and (g)) better reveals the underlying configuration of the magnetic domains, as evidenced by the morphology of flux penetration. It is interesting to note that the stripe domains in the Py layer do not only act as channels for the smooth and isothermal flux penetration occurring at high temperatures, but also influence the dynamics of flux avalanches of thermomagnetic origin at low temperatures. This is shown in panels (d) and (h) obtained at *T* = 4 K after applying *μ*_0_*H* = 3 mT. Smaller avalanches are prevented from penetrating into the Py layer, resulting in some flux accumulating at the interface. The avalanches entering the Py tend to align with the direction of the magnetic domains, resulting in a bending of their trajectories at the interface, in order to follow the easiest path towards the center of the Py. Moreover, the magnetic field on top of the avalanches is weaker compared to the bare Nb film. This effect is attributed to the high magnetic permeability of the Py layer which, as in *μ*-metal screening sheets, favours the diffusion of magnetic field inside the Py layer.

### Imprinting vortex footsteps in a thin Py layer

In the previous section we have shown that the magnetic landscape produced by a thick Py layer shapes flux penetration in the superconductor. Attempting to revert this scenario, so that the superconducting flux rules over the magnetic template, can be achieved by reducing the volume of the magnet in such a way to decrease its associated magnetic energy.

**Figure 5:**
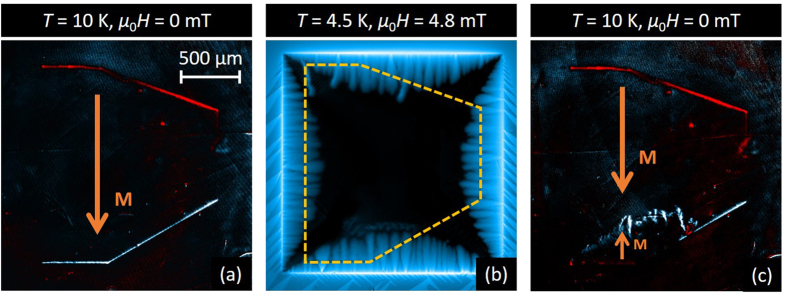
In-plane magnetisation reversal induced by smooth flux penetration in the Nb. MO images of a Nb film with a thin (50 nm) Py layer after applying an in-plane field of 10 mT along a direction close to the vertical axis. (**a**) Image at *T* = 10 K > *T*_c_. The white (red) rims evidence the positive (negative) stray field at the borders of the Py layer. (**b**) MO image obtained at *T* = 4.5 K in the smooth flux penetration regime for an applied field *μ*_0_*H* = 4.8 mT pointing out of the image. After obtaining the image (**b**), the temperature is increased to *T* = 10 K and *μ*_0_*H* = 0 mT, resulting in the image in panel (**c**). Panel (**c**) shows clear reversal of the in-plane magnetisation induced by flux penetration.

Figure 5(a) shows a MO image of the thin Py layer after applying an in-plane field of 10 mT at *T* = 10 K > *T*_c_. As pointed out above, the net in-plane magnetic moment of the thin film is about 6 times weaker than for the 460 nm-thick Py layer. Panel (b) shows the smooth flux penetration in the superconductor at *T* = 4.5 K for *μ*_0_*H* = 4.8 mT. As expected from the radically different magnetic domain landscape featured in the MFM images in Fig. 2, there is no flux guiding inside the thin Py layer, in sharp contrast to the behavior observed in the thick Py layer. Moreover, due to the weaker value of *B*_*z*_ estimated from Fig. 2(f), the asymmetry in flux penetration in the thin Py is less pronounced, although a slight delay for the vortex entrance can still be observed at the white Py side, populated by antivortices. There is no noticeable influence on flux penetration at the borders perpendicular to the in-plane magnetisation.

Strikingly, when the temperature is increased up to 10 K and *H* is subsequently decreased to zero (Fig. 5(c)), we observe that smooth flux penetration in the Nb layer has left a clear imprint in the Py layer. This imprint corresponds to a reversal of the in-plane magnetic domains as indicated by arrows in Fig. 5(c). Note that smooth flux penetration leaves no traces at the other borders of the Py layer. In order to account for this effect, we start by recalling that 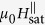 is smaller than 2 mT, meaning that the magnetic state of the Py layer can be easily switched by an in-plane field^31^. Considering that the flux front advancing in the Nb layer is made of vortices which generate an in-plane magnetic field component at the Py layer, we wonder if that field component is large enough to switch the in-plane magnetic moment. In the case of a single Pearl vortex^37^ in Nb, it is easy to calculate^5^ that the in-plane field component at a distance *z* = 10 nm from the top surface of the superconductor reaches a maximum value ~10 mT at a distance *r* ~ *λ* from the vortex center and decays as 1/*r*^2^, producing a significant *B*_||_ even at a few hundreds of nanometres from the vortex core. We can then safely assume that this in-plane magnetic field will be even reinforced by the joint contributions of the vortices located in the vicinity of the superconducting flux front in our sample. This discussion leads us to conclude that *B*_||_ at the flux front is strong enough to flip locally the magnetic moments of the Py layer. This argument naturally explains why the flux penetration does not leave imprints at the other borders of the Py layer where *B*_||_ is either in the same direction or perpendicular to the original magnetic moment orientation.

**Figure 6:**
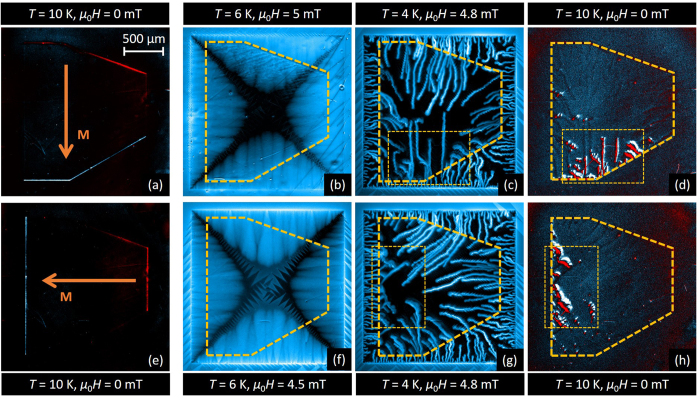
Interaction between a 50 nm-thick Py layer and a Nb film. MO images of the Nb film with a 50 nm-thick Py layer on top, after applying an in-plane field of 10 mT close to the vertical (upper row) and horizontal (lower row) directions. First column: images obtained at *T* = 10 K > *T*_c_. The white (red) rims evidence the positive (negative) stray field at the borders of the Py layer. Second column: MO images obtained at *T* = 6 K in the smooth flux penetration regime, for an applied field pointing out of the images, (**b**) *μ*_0_*H* = 5 mT and (**f**) *μ*_0_*H* = 4.5 mT. Third column: MO images corresponding to *T* = 4 K and *μ*_0_*H* = 4.8 mT in the abrupt flux penetration regime, where flux avalanches of thermomagnetic origin are observed. Last column: images obtained at *T* = 10 K > *T*_c_ and *μ*_0_*H* = 0, starting from the state in the third column. The remanent state of the Py layer shows clear imprints of flux avalanches. The yellow dashed rectangles mark the area studied more carefully in Fig. 7.

Figure 6 summarizes the results obtained for a 50 nm-thick Py layer. The first column (panels (a) and (e)) corresponds to the MOI recorded above *T*_c_ and showing the stray field of the thin Py layer, estimated to be around 0.35 mT in the garnet.

The second column of Fig. 6 shows smooth flux penetration occurring at 6 K for *μ*_0_*H* = 5 mT (panel (b)) and *μ*_0_*H* = 4.5 mT (panel (f)). In sharp contrast to the highly anisotropic flux penetration observed for the thick Py layer, now the flux front morphology is very similar to an uncovered Nb film. As we discussed above (see Fig. 2(b)), for this sample, there are no domains with out-of-plane magnetisation to impede or guide the motion of vortices.

At lower temperatures, within the thermomagnetic instability regime, flux avalanches undergo a deflection of their trajectories when penetrating the area covered by the Py layer (Fig. 6(c,g)). Similar behaviour has been observed in superconductor/metal hybrid systems^2^,^3^8–40 and attributed to the eddy currents induced in the thick metallic layer. However, in the present case, the thickness of the Py layer is too small to induce avalanche deflection and therefore another deflection mechanism needs to be invoked. Likely the source of this deflection has a magnetic origin, since avalanches tend to align with the magnetisation direction. The cause of the observed preferential direction for the avalanche propagation lies in the energy needed to switch the in-plane magnetic moment. Indeed, avalanches propagating parallel to the in-plane magnetic moment do not induce in-plane *M* flipping, whereas avalanches propagating antiparallel to the in-plane moment are damped by the energy they spent in inducing in-plane *M* flipping. A closer inspection of Fig. 6(c,g) indeed shows that avalanches entering from the red side of the Py tend to advance more easily following the direction of the magnetisation, whereas avalanches entering from the white side penetrate less under the Py layer. The smoking gun evidence for this interpretation comes from the last column of Fig. 6.

Starting from the state presented in the third column of Fig. 6, we then increased the temperature above *T*_c_ and recorded the resulting magnetic landscape as shown in Fig. 6(d,h). We observe the presence of a clear magnetic signal corresponding to traces left by flux avalanches, but mainly on the side where the flux has been forced to switch the in-plane magnetic moment of the Py layer. These imprints of avalanches were seldom visible in the 460 nm-thick permalloy layer, while they were also observed in the 100 nm-thick Py. This seems to point at the importance of the magnetic pattern of the Py layer in order to print the magnetic flux, and especially at the crucial role played by interfaces between domains with different in-plane magnetisations. These imprints seem to correspond to head-to-head Bloch domain walls pointing up and down, and delimiting interfaces between regions of different **M**, as also observed in ref. 31.

Even though the signal is stronger on the white side of the Py, weak traces induced by the vortex avalanches can be seen on the whole surface of the magnetic layer. These types of traces are formed by pairs of lines with opposed polarity as observed in Fig. 6(d,h). For a given initial **M**, these traces always respect the same polarity through the whole magnetic layer, i.e. dark stripe on one side of the trace, bright stripe on the other side. This type of magnetic structure is the same as the pattern observed by Uspenskaya *et al*.^31^ in similar hybrid structures and are correlated with the direction of **M**. They were identified as Néel domain walls, whose footprints are dark and bright stripes produced by the local poles appearing at both sides of the in-plane **M** inside the wall.

**Figure 7:**
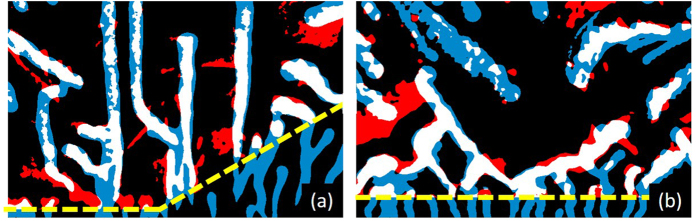
Spatial correlation between flux avalanches and their imprints in the 50 nm-thick Py layer. Binary images of the region marked by a yellow dashed rectangle (**a**) in Fig. 6(c,d), and (**b**) in Fig. 6(g,h). The superposition of flux avalanches (in blue) and their negative field imprints (in red) is represented in white.

In order to highlight the correlation between flux avalanches and their imprints in the magnetic layer, we will now focus on certain particular areas of the sample, marked by a yellow dashed rectangle, for each series of measurements shown in Fig. 6. Figure 7(a,b) shows the superposition of two binary images, where low fields were represented in black and high field were coloured. Flux avalanches at 4 K represented in Fig. 6(c,g) are painted in blue, while their negative field imprints at 10 K featured in Fig. 6(d,h) are in red. The reason for this choice is that, surprisingly, avalanches happen to be spatially strongly correlated to the stripes with stray field opposed to the applied field, as shown by the regions coloured in white, where avalanches and their imprints overlap. Strikingly, the imprints in the magnetic layer reveal an excellent correlation with the position of the flux avalanches. Moreover, the magnetic imprints left by avalanches could even be maintained and observed up to 300 K, opening the path to a new way to visualize flux avalanches *ex-situ*. Indeed, much like magnetic particles highlight the flux pattern in the Bitter decoration technique, the magnetic layer can be used here to record avalanches and observe them at temperatures well over *T*_c_. In the remainder of this paper, we will study the room temperature imprinting of a hard ferromagnetic sample in the thick and thin Py to shed light on the different behaviour of the two layers under these conditions.

### Room temperature imprinting of magnetic patterns in Py layers

**Figure 8:**
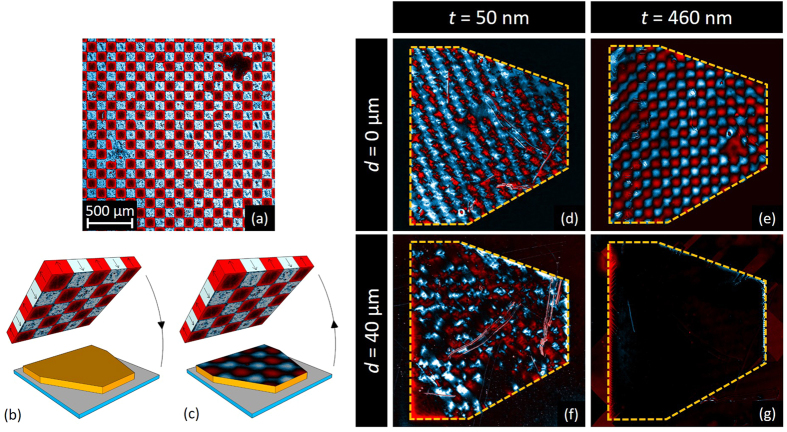
Room temperature imprinting of an inhomogeneous magnetic landscape in the Py layers. (**a**) MO image of a NdFeB magnetic pattern with 100 × 100 *μ*m^2^ domains alternating up (white) and down (red) magnetisation. This pattern is then (**b**) laid upside down against the Py layer and (**c**) removed from the Py, leaving an imprint of the magnetic template in the magnetic layer. Images in (**d**,**e**) show that this procedure is efficient to print the magnetic pattern in the 50 nm-thick Py as well as in the 460 nm-thick. When a 40 *μ*m pergamine sheet is inserted between the magnetic pattern and the Py during the transfer, the printing is still observed in the thin Py film, while it is completely absent in the thick layer (panels (**f,g**)).

In order to unambiguously demonstrate that imprinting of an inhomogeneous magnetic field landscape is possible in the thin Py layer even at room temperature, we use a strong source of magnetic field consisting of a 3 *μ*m-thick hard ferromagnetic NdFeB film with a chessboard pattern consisting of alternating domains with up and down out-of-plane magnetisation, as shown by the MO image in Fig. 8(a). Details of sample fabrication for obtaining stable patterns of this type can be found in the Methods section and in ref. 41. The magnetic field at a distance of 26 *μ*m from the surface was measured in a similar sample by scanning Hall probe microscopy, giving values varying between +10 and −13 mT, depending on the position^41^. Therefore, the maximum magnetic field in the vicinity of the sample surface is expected to reach values of several hundreds of mT.

Before all imprintings, the magnetic history of the Py layers was first erased by applying an in-plane magnetic field of 20 mT. In practice, imprinting of the chessboard pattern was obtained by approaching the NdFeB sample upside down to the Py layer, as schematically shown in Fig. 8(b). The chessboard pattern was then removed (Fig. 8(c)), leaving an imprint that was observed by magneto-optical imaging. The resulting imprints after the Py layer and the NdFeB have been in contact (*d* = 0 *μ*m) are shown in Fig. 8(d,e) for the 50 nm-thick and 460 nm-thick Py layers, respectively. In both cases, the imprints of the chessboard pattern are very clear. This may seem in contradiction with the low temperature results for the thick Py layer, where we could not record flux avalanches. However, it should be stressed that the magnetic field generated by the NdFeB pattern is several dozen times larger than the field of an avalanche, which is roughly a few dozens of mT^42^. Therefore, the field is high enough to change the microscopic domain distribution inside the thick Py layer, leading to domains with significant out-of-plane remanent magnetisation.

If we repeat the printing process, but now insert a 40 *μ*m-thick piece of pergamine paper between the Py layer and the NdFeB chessboard pattern, we obtain the images shown in Fig. 8(f,g). Strikingly, the magnetic imprint is completely absent from the 460 nm-thick Py layer, while it is still visible in the 50 nm-thick Py. This clearly pinpoints the main difference in the behaviour of the two Py layers with respect to magnetic printing: high out-of-plane fields are necessary to leave a mark in the thick Py, where domains with out-of-plane magnetisation exist, whereas weak in-plane fields are sufficient to imprint magnetic patterns in the thin Py, by flipping the in-plane magnetisation locally.

In summary, we have experimentally demonstrated the possibility to visualise *ex situ* both smooth and abrupt magnetic flux penetration in a Nb film, by depositing a thin Py layer on top. The imprints left by the flux seem to be made of Bloch type domain walls separating head-to-head in-plane magnetic domains. We show that flux avalanches progress less when they are forced to invert the polarization of the Py layer as compared to the case where no magnetisation switching occurs. We attribute this effect to the work needed to revert the polarization orientation, but other possible damping mechanisms should be considered, as for instance the generation of magnons^43^ or the fact that vortex avalanches may be limited by the much slower Walker velocity of propagation of magnetic domain walls^44^. The method here introduced is actually not limited to thin Py layers, although much larger fields are needed to leave imprints in thick Py layers. In addition, thick Py layers tend to severely modify flux propagation in the superconducting film and this technique could therefore no longer be considered as non invasive. The undeniable appeal of the technique lies in its simplicity and the possibility to explore high *T*_c_ superconductors. We expect to trigger further experimental and theoretical studies to discover new magnetic compounds optimizing the resolution of the technique down to single vortex imprints.

## Methods

### Nb/Py hybrid fabrication

The 140 nm-thick Nb films covered by a 5 nm-thick SiO_2_ layer were prepared in a home-built electron beam UHV evaporator. More information about the Nb film fabrication can be found in ref. 45. The Py layers were deposited in an Alliance Concept RF/DC sputtering machine. The base pressure in the introduction chamber was 10^−7^ mbar. To improve the sticking of the Py on top of the SiO_2_, a 3 nm-thick Ta buffer layer was first deposited using DC sputtering with the following parameters: power of 40 W, 20 sccm argon flux and 1.3 Å /s deposition rate at 100 mm distance. The permalloy layer is composed of 81% Ni and 19% Fe and was deposited with 20 W power and 20 sccm argon flux. The sputtering of the 50 nm-thick (460 nm-thick) Py was done at a distance of 100 mm (50 mm) with a rate of 1.3 Å/s (5.2 Å/s). The magnetic anisotropy in the Py layer comes from the magnetron field.

### Thermomagnetically patterned NdFeB sample fabrication

The chessboard patterned magnetic sample is a 3 *μ*m-thick NdFeB layer deposited by high rate triode sputtering on a thermally oxidised 100 mm Si wafer covered by a 100 nm-thick buffer layer of Ta. A capping layer of 100 nm-thick Ta was also deposited on top of the NdFeB to avoid oxidation. Magnetisation was reversed over 1.2 *μ*m in 100 × 100 *μ*m^2^ regions by local heating with a laser in an external field, forming a chessboard pattern of alternating domains with up and down magnetisation. The coercive field is around 1.9 T at 300 K. More details about sample fabrication can be found in ref. 41.

### SQUID magnetometry

Magnetisation measurements were obtained in a Quantum Design SQUID magnetometer. From the temperature dependence of the upper critical field, *H*_c2_(*T*), we have estimated the superconducting coherence length *ξ*(0) = 9.7 nm.

### Magnetic force microscopy (MFM)

Magnetic Force Microscopy (MFM) images were obtained in a Multimode AFM system (Digital Instruments Nanoscope III - VEECO) in interleave tapping mode (scan lift of 20 nm) with a SSS-MFMR probe from NANOSENSORS, magnetized with an external magnet. The RMS roughness of the Py films is 0.83 ± 0.16 nm in a 10 × 10 *μ*m^2^ area.

### Magneto-optical imaging (MOI)

Magneto-optical imaging is a technique based on the Faraday rotation of linearly polarized light in a 3 *μ*m-thick Bi-doped yttrium iron garnet (Bi:YIG) with in-plane magnetic domains, placed on top of the investigated hybrid sample. The Faraday-active material, deposited on a Gd_3_Ga_5_O_12_ substrate, receives a top layer of 100-nm thick Al serving as a mirror. The sensor is mounted upside down, with the Al mirror in close contact with the sample under study. Since the rotation of polarisation is proportional to the local magnetic field *B*_*z*_ in the indicator, the use of an analyser oriented perpendicularly to the initial direction of polarisation results in images where the intensity is proportional to *B*_*z*_. Images are acquired with a CCD camera RETIGA-4000, and each pixel in the images corresponds to an area of 1.618 × 1.618 *μ*m^2^. Post-image processing of the images was done to remove the inhomogeneous illumination and field-independent background, using the ImageJ software. Calibration of the MOI was done following a similar procedure as in ref. 46. Both liquid He and closed-cycle cryostats were used to cool down the samples. The external magnetic field was applied through a current fed cylindrical coil. More details about the set-up can be found in ref. 45,47.

### Numerical simulations

Numerical simulations were performed using the COMSOL Multiphysics software.

## Additional Information

**How to cite this article**: Brisbois, J. *et al*. Imprinting superconducting vortex footsteps in a magnetic layer. *Sci. Rep.* **6**, 27159; doi: 10.1038/srep27159 (2016).
